# Adaptive Surround Modulation of MT Neurons: A Computational Model

**DOI:** 10.3389/fncir.2020.529345

**Published:** 2020-10-26

**Authors:** Parvin Zarei Eskikand, Tatiana Kameneva, Anthony N. Burkitt, David B. Grayden, Michael R. Ibbotson

**Affiliations:** ^1^Department of Biomedical Engineering, The University of Melbourne, Parkville, VIC, Australia; ^2^Faculty of Science, Engineering and Technology, Swinburne University of Technology, Hawthorn, VIC, Australia; ^3^National Vision Research Institute, Australian College of Optometry, Carlton, VIC, Australia

**Keywords:** vision, neural model, motion perception, middle temporal (MT), adaptive surround modulation

## Abstract

The classical receptive field (CRF) of a spiking visual neuron is defined as the region in the visual field that can generate spikes when stimulated by a visual stimulus. Many visual neurons also have an extra-classical receptive field (ECRF) that surrounds the CRF. The presence of a stimulus in the ECRF does not generate spikes but rather modulates the response to a stimulus in the neuron's CRF. Neurons in the primate Middle Temporal (MT) area, which is a motion specialist region, can have directionally antagonistic or facilitatory surrounds. The surround's effect switches between directionally antagonistic or facilitatory based on the characteristics of the stimulus, with antagonistic effects when there are directional discontinuities but facilitatory effects when there is directional coherence. Here, we present a computational model of neurons in area MT that replicates this observation and uses computational building blocks that correlate with observed cell types in the visual pathways to explain the mechanism of this modulatory effect. The model shows that the categorization of MT neurons based on the effect of their surround depends on the input stimulus rather than being a property of the neurons. Also, in agreement with neurophysiological findings, the ECRFs of the modeled MT neurons alter their center-surround interactions depending on image contrast.

## Introduction

The classical receptive field (CRF) of a spiking visual neuron is defined as the region in the visual field within which the presentation of a visual stimulus can generate spikes in that neuron. Many visual neurons also have an extra-classical receptive field (ECRF) that surrounds the CRF. The presence of the stimulus in the ECRF cannot generate a response by itself but can have an excitatory or inhibitory effect on the neuron's response (Barlow and Levick, 1965). For direction-selective neurons, the directional tuning of the surround is mainly antagonistic, which results in a reduction of the activity of the neuron when the motion in the surround is in the preferred direction of the center; this is called an antagonistic surround. However, in some neurons, the surround can instead have a facilitatory effect, which reinforces the activity of the neuron when the motion in the surround is in the preferred direction of the center; this is a facilitatory surround (Barlow and Levick, 1965).

It is commonly reported that most of the neurons in the primate middle temporal (MT) area, which is a motion specialist region, have directionally antagonistic surrounds (Albright, 1984). However, Huang et al. (2007) showed that the dominance of the antagonistic surround in the literature is due to the characteristics of the stimuli that have been commonly used.

Here, we present a model in which the ECRFs of MT neurons are adaptive to the changes in the input stimulus. The surrounds have an antagonistic effect when there is a discontinuity in the input stimulus. In our definition, any inconsistency in the characteristics of the stimulus are regarded as discontinuities, including changes in luminance or shape. We show in our model that the level of the antagonism driven by the surround increases with increases in stimulus contrast. The antagonistic effect of the surround assists the MT neurons to detect discontinuities in the input stimulus and segregates stimuli that are moving in different directions. The antagonistic surrounds of the MT neurons switch to having facilitatory effects when there is coherency in the input stimulus, which facilitates the propagation of integrative signals.

Existing neurophysiological findings support the concept of stimulus-dependent center-surround characteristics (Thiele, 2007). To demonstrate alterations in surround modulation that depend on the input stimulus, Huang et al. (2007) evaluated the activity of MT neurons in response to two different stimuli and observed adaptive changes of the surround effect. According to their observations, the surround represents integrative features in response to moving contours to overcome ambiguous motion information resulting from the aperture problem. The aperture problem refers to the generation of ambiguous motion information by neurons with small receptive fields that are only able to measure the motion component in the direction orthogonal to the long edge of a bar stimulus (Born and Bradley, 2005). However, the surround regions of Huang et al. (2007) MT neurons show antagonistic features in response to moving dot fields that carry unambiguous motion signals. These experiments demonstrate switching of antagonistic surrounds to integrative surrounds when there is uncertainty in the CRF (Huang et al., 2007). The source of motion uncertainty could be the aperture problem, low luminance contrast, or the presence of noise. It is possible to make similar conclusions based on the results of experiments performed by Pack et al. (2005), who observed an enlargement in the spatial summation of the receptive fields of MT neurons with decreasing stimulus contrast. The increases in the sizes of the receptive fields are equivalent to the characteristics of the integrative surround. Therefore, a reduction in the contrast of the stimulus, which contributes to the uncertainty in the center of the receptive field, results in strengthening of the integrative features of the surround compared to the case at high contrasts (Pack et al., 2005).

Huang et al. (2008) also showed that the modulatory effects of MT neurons depend on the response magnitudes of the MT neurons to different stimuli. Their experiments showed that a stimulus that drives low amplitude responses produces surround integration while a stimulus that drives high amplitude responses produces surround antagonism and, therefore, image segmentation.

Pack et al. (2005) investigated the effects of changes in stimulus contrast on the suppressive surrounds of the neurons in area MT. They found a reduction in the suppressive influence of the surround in some MT neurons at low contrasts. The responses of neurons continuously increase with increases in the size of the stimulus, without saturating at low contrasts. For high contrasts, responses increase with increasing diameter until an optimum is reached, and then fall as the diameter expands further. Therefore, the responses of the neurons to stimuli with large diameters at high contrasts are significantly lower than the responses of the same neurons to the same large-diameter stimuli at low contrasts (Pack et al., 2005).

The results of psychophysical experiments performed by Tadin et al. (2003) can be also explained by the possibility of the facilitation of MT responses at low contrasts with larger stimuli. They proposed that the transition from suppressive surround to integrative surround occurs at around 5% contrast (Tadin et al., 2003). In their psychophysical experiments, they measured the duration threshold, which is the time that human observers need to recognize the accurate direction of motion. They observed an increase in duration threshold with increases in contrast and *vice versa*. They also revealed an increase in duration threshold as the size of the moving stimulus increased when embedded in high contrast noise, which decreased the visibility of the stimulus (Tadin et al., 2003).

In agreement with these neurophysiological and psychophysical findings, the MT neurons in our model have stimulus-dependent modulatory effects. The surrounds of the MT neurons have an inhibitory effect in the case of discontinuities in the stimulus or a high level of the contrast and a facilitatory effect where there is a coherency in the stimulus. Our proposed model explains the circuitry of this modulatory effect of the surround in response to the properties of the input stimulus.

## Methods

There is a clear distinction between two different categories of neural models, functional and mechanistic models (Kay, 2018). Functional models account for the functional relationship between input, namely the stimulus, and output, which are the responses of the neurons. However, mechanistic models go further and not only predict the response of the neurons but also include the mechanism that associates the relationship between the stimulus and the responses of the neurons (Kay, 2018). The proposed model here is a mechanistic model that describes the relationship between different types of neurons in V1 and MT by explaining the specific role of every subtype of the modeled elements in the estimation of the correct direction of motion, which accords to the existing neurophysiological data.

**Figure 1:**
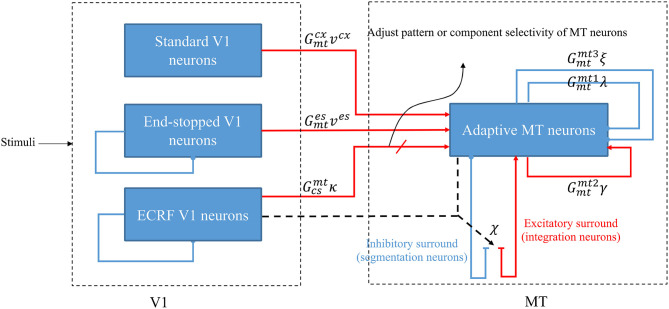
A schematic diagram showing the interconnections of the neurons in MT and V1. Red arrows represent excitatory interconnections between neurons and blue lines indicate inhibitory connections. Black solid lines indicate the effect of ECRF V1 neurons on the threshold level defined by the activity of end-stopped neurons for the inhibitory connections between them. Black dashed lines show the adaptive center-surround interactions between MT neurons that depend on the activity of ECRF neurons, which are expressing the changes in the contrast of the input stimulus. Depending on the input received from these V1 neurons, the surround characteristics switch between antagonistic and integrative or, a null surround, which is something between these features. The level of the excitatory input from V1 neurons with suppressive surrounds that are sensitive to the luminance of the stimulus determines the pattern or component motion selectivities of MT neurons.

The model is a two-stage process with initial visual information extracted by neurons that model the responses of V1 cells and then transmit this information to the neurons in MT for integration of local motion signals and segregation of overlapping stimuli that are moving in different directions (Figure 1). There are models of three types of neurons in V1 that each have distinctive features and different roles in visual information processing: standard complex V1 neurons, end-stopped V1 neurons, and V1 neurons with ECRFs, which have surrounds that are sensitive to the luminance of the stimulus and suppress center responses (Zarei Eskikand et al., 2018).

Standard V1 neurons determine the borders of the stimulus (edges), end-stopped neurons respond only to the unambiguous motion information of the end-points of the stimulus, and ECRF V1 neurons transmit initial form information to the next area to overcome the illusion formed at the crossing junctions of overlapping stimuli. The initial motion and form information are prosessed by the MT neurons in the next stage (Zarei Eskikand et al., 2018, 2019). A summary of different types of neurons in the model is presented in Table 1. The size of the receptive fields in Table 1 considers only the center of the receptive fields of the neurons and does not include the surround of the receptive fields. In contrast to the previous computational models by Zarei Eskikand et al. (2018, 2019), where there are two different types of MT neurons—integration and segmentation neurons—the model presented here introduces only a single type of MT neuron that has adaptive center-surround interactions depending on the characteristics of the input stimulus. Therefore, the categorization of MT neurons into segmentation and integration neurons depends on the input stimulus rather than being a property of the neuron. The modulatory surrounds of the MT neurons, which accord with existing neurophysiological data, improve the flexibility of the modeled neurons in response to changes in stimuli. The observation that this change in center-surround interaction depends on contrast suggests that the strategy of the visual system is to integrate motion signals when they are weak, thereby increasing sensitivity to areas of the image with low contrast (Pack et al., 2005).

**Table 1:** Different types of the neurons in the model and their functions, and receptive field (RF) sizes.

	**Type**	**Definition/Role**	**Direction selective**	**Orientation selective**	**Contrast sensitive**	**Size of the RF**
V1	Complex V1 neurons	Extract initial motion information. Determine the borders of the stimulus.	YES	YES	NO	1°
	End-stopped neurons	Respond only to the motion information of the end-points of the stimulus to overcome aperture problem.	YES	YES	NO	1°
	ECRF V1 neurons	Extract initial form information. Differentiate the intrinsic terminators from extrinsic terminators in the case of overlapping stimuli.	NO	YES	YES	1°
MT	MT neurons	Integration of local motion signals to estimate the direction of motion and segregation of overlapping stimuli that are moving in different directions.	YES	YES	YES	7°

This proposed relationship between neurons in the model creates the potential to easily extend the model to cover other areas of the visual cortex and explain many of the neurophysiological findings in relation to the motion of different stimuli.

### V1 Neurons

Initial motion information is extracted by standard complex V1 neurons that have small receptive fields. The model of standard complex V1 neurons is based on the motion energy filter, which is a modified version of the Reichardt motion detector (Adelson and Bergen, 1985; Van Santen and Sperling, 1985). The receptive fields of these neurons are spatiotemporal filters. The spatial filter of these motion detectors is modeled by a Gabor function and the temporal filter is a multi-stage low pass filter modeled by

(1) $$\begin{array}{l} g_{n}\left(t\right)={\left(\frac{t}{\tau_{g}}\right)}^{n}\exp\left(-\frac{t}{\tau_{g}}\right)\left[\frac{1}{n!}-\frac{{\left({\frac{t}{\tau }}_{g}\right)}^{2}}{\left(n+2\right)!}\right], \end{array}$$

where τ_*g*_ is the time constant of the filter and *n* takes values of 6 and 9, simulating the delay between two different temporal filters to compute the motion of the stimulus. The neurons are selective to eight different directions (Zarei Eskikand et al., 2016). The value of the parameters of the model are listed in Table 2.

**Table 2:** The constant parameters used in the model, their values, and their units.

**Description**	**Parameter**	**Value**
Connection strength of input to the end-stopped neurons	$G_{es}^{cx1}$	2
Connection strength of inhibitory connections on end-stopped neurons	$G_{es}^{cx2}$	3
Connection strength of complex V1 neurons to MT neurons	$G_{mt}^{cx}$	0.5
Connection strength of end-stopped V1 neurons to MT neurons	$G_{mt}^{es}$	1
Connection strength of center-surround V1 neurons to component MT neurons	$G_{cs}^{mt}$	2
Connection strength of excitatory connections to MT neurons	$G_{mt}^{mt2}$	0.1
Connection strength of inter-directional inhibitory connections	$G_{mt}^{mt1}$	1
Connection strength of long-range inhibitory connections	$G_{mt}^{mt3}$	1
Spatial extent of the inhibitory connections defined by the number of the neighboring neurons	ϕ	6
Number of neurons at each location-selective to different directions	*N*	8
Constant value for the threshold on the activity of complex V1 neurons	ρ^*cx*^	0.13
Spatial extent of the surround of MT neurons	M	49
The threshold on the changes in the contrast level	*c*^*r*^	0.2
The slope of changes in the level of suppression with contrast	a	−10
The slope of changes in the level of suppression with the activity level of the neurons with different direction selectivity	b	2.4
The limitation constant on the level of the surround suppression	k	2
Constant value for the threshold on the activity of MT neurons	*T*^*mt*^	0.001
Constant value for the threshold on the activity of end-stopped neurons	ρ^*es*^	0.3
Decay rate of the activity of MT neurons	τ_*mt*_	0.01
Decay rate of the activity end-stopped neurons	τ_*es*_	0.01
Simulation time step	Δ*t*	0.01
Time constant of the temporal filter	τ_*g*_	0.01
Time delay of inhibition between MT neurons	*T*^*in*^	0.4
Spatial frequency	*f*	1.1
Standard deviation of horizontal spatial Gaussian filter	σ_*x*_	0.5
Standard deviation of vertical spatial Gaussian filter	σ_*y*_	0.5
Standard deviation of center portion of horizontal spatial Gaussian filter	σ_*x*_*c*_	0.35
Standard deviation of center portion of vertical spatial Gaussian filter	σ_*y*_*c*_	0.4
Standard deviation of surround portion of horizontal spatial Gaussian filter	σ_*x*_*s*_	0.4
Standard deviation of surround portion of vertical spatial Gaussian filter	σ_*y*_*s*_	0.5
Strength of center portion of spatial Gaussian filter	*A_*c*_*	1
Strength of surround portion of spatial Gaussian filter	*A_*s*_*	0.72

Standard complex V1 neurons detect only local motion signals because of their small receptive fields, which results in the aperture problem. However, the neurons at the terminators (corners) of the stimulus provide unambiguous motion signals because the corners are two-dimensional. To suppress the ambiguous activity of standard complex V1 neurons along the long-edges, a model of end-stopped V1 neurons is essential, which respond only to the terminators of the stimulus (Hubel and Wiesel, 1965; Pack et al., 2003). The activity of end-stopped V1 neurons is modeled using inhibitory interconnections between neurons. This inhibition is effective only if the neighboring neurons have activity above a threshold level (Tsui et al., 2010). The activity of these neurons is modeled by

(2) $$\begin{array}{r} \frac{d}{dt}v_{x,y,\theta }^{es}\left(t\right)=\left(1-v_{x,y,\theta }^{es}\left(t\right)\right)\left(G_{es}^{cx1}v_{x,y,\theta }^{cx}\left(t\right)\right)\\ -v_{x,y.,\theta }^{es}\left(\tau_{es}+G_{es}^{cx2}\Gamma_{x,y,\theta }\left(t\right)\right), \end{array}$$

where $v_{x,y,\theta }^{es}$ is the activity of an end-stopped neuron selective to direction θ (eight different directions: 0°, 45°, 90°, …) located at the coordinate (*x,y*), $v_{x,y,\theta }^{cx}$ is the activity of the standard complex neuron in the same location and direction, τ_*es*_ is a decay rate, and $G_{es}^{cx1}$ and $G_{es}^{cx2}$ are constant gains. The activities of the standard complex V1 neurons are the outputs of the motion energy filters, which are computed from the correlation of the input image with the spatiotemporal filters. The parameter Γ_*x,y*,θ_ is the inhibition that the end-stopped neuron receives from standard complex V1 neurons when the activity of the neighboring standard complex neurons is above the threshold, $\rho_{x,y}^{cx}$,

(3) $$\begin{array}{l} \Gamma_{x,y,\theta }=\{\begin{array}{c} \sum_{i=-8}^{8}\sum_{j=-8}^{8}\mu_{i,j}v_{x+i,y+j,\theta }^{cx}\;v_{x+i,y+j,\theta }^{cx}{|}_{i=-3,j=-3}^{i=3,j=3}>\rho_{x,y}^{cx}\\ 0\;Otherwise,\; \end{array} \end{array}$$

where μ_*i,j*_ is the inhibitory connectivity matrix that extends across a patch of 8x8 neighboring neurons and has a discretized Gaussian shape (Zarei Eskikand et al., 2016). These lateral inhibitory connections between standard complex V1 neurons (in our model) result in the end-stopping feature. In our proposed scheme, there are no interactions from the neurons that have end-stopping features upon those that do not have this feature (complex V1 neurons).

The activities of end-stopped neurons at the intrinsic terminators, the actual end-points of the stimulus, carry unambiguous motion information, while the direction perception of the motion by end-stopped neurons at the extrinsic terminators, formed when objects overlap, conflicts with the global movement of the stimulus.

Apart from the standard complex V1 neurons and end-stopped neurons, there is another set of V1 neurons in our model (ECRF neurons) that play a significant role in estimating the accurate direction of movement of the objects when they are overlapped. These modeled neurons have a high level of activity at the intrinsic terminators and their activities are strongly inhibited at the extrinsic terminators because of the stimulation of their suppressive surrounds by the border of the stimulus. To consider the suppressive surround of the neurons, the receptive field is modeled as a difference of Gaussians (DoG),

(4) $$\begin{array}{l} R_{x,y,o}=A_{C}\exp\left(-\left(\frac{x_{o}^{2}}{\sigma_{xc}^{2}}+\frac{y_{o}^{2}}{\sigma_{yc}^{2}}\right)\right)-A_{S}\exp\left(-\left(\frac{x_{o}^{2}}{\sigma_{xs}^{2}}+\frac{y_{o}^{2}}{\sigma_{ys}^{2}}\right)\right), \end{array}$$

where (*x*_*o*_, *y*_*o*_) are oriented coordinates with orientation *o* at spatial location (*x*, *y*). These oriented coordinates result in the orientation selectivity of the receptive fields of these neurons. The parameters $\sigma_{xc}^{2}$ and $\sigma_{yc}^{2}$ are the standard deviations of the centers of the receptive fields, $\sigma_{xs}^{2}$ and $\sigma_{ys}^{2}$ are standard deviations of the surround, and *A*_*C*_ and *A*_*S*_ are constants.

The defined model of the receptive field of the neurons is applied on the stimulus to compute initial form information. The activity levels of standard complex V1 neurons are gated by the activity of ECRF neurons such that the resulting standard complex V1 outputs have a high level of motion information only when ECRF neurons are active, which occurs at the intrinsic terminators (Zarei Eskikand et al., 2018).

### MT Neurons

The MT neurons in the model are adaptive to changes in the contrast of the stimulus as the center-surround interaction of MT neurons depends on the contrast of the input stimulus. The neurons have an integrative surround in response to input motion coherence when the motion of the center is in the same direction as the motion of the surround. However, this integrative surround can switch to an antagonistic surround when there is a discontinuity in the input stimulus that results in the suppression of its activity.

MT neurons receive excitatory input from standard V1 and end-stopped neurons. The gain of the excitatory input of the end-stopped neurons is higher than the activity of the complex V1 neurons to help deal with the aperture problem. The activity of MT neurons is also enhanced by the received input as the result of the interaction of form and motion information, which is computed by the activity of standard complex V1 neurons, gated by ECRF neurons. This excitatory input is essential to strengthen the influence of the intrinsic terminators compared to the extrinsic terminators. MT neurons receive excitatory connections from their neighboring neurons to propagate the unambiguous activity from the terminators along the whole of the object. The level of the excitatory connection, λ_*x,y*,θ_, is gated by the summation of the activity of ECRF neurons, $v_{x,y,o}^{cs}$, over different orientations, and it is computed as

(5) $$\begin{array}{l} \lambda_{x,y,\theta }=\{\begin{array}{c} \sum_{i=-3}^{3}\sum_{j=-3}^{3}v_{x+i,y+j,\theta \;}^{mt}v_{x+i,y+j,\theta }^{mt}-v_{x,y,\theta }^{mt}>T_{x,y}^{mt}\\ and\;\sum_{o}v_{x,y,o}^{cs}>0\\ 0\;otherwise, \end{array} \end{array}$$

where $v_{x,y,\theta }^{mt}$ is the activity level of the MT neuron selective to direction θ at location (*x, y*). There is an inter-directional inhibition between MT neurons at the same location, γ_*x,y*,θ_ computed as

(6) $$\begin{array}{l} \gamma_{x,y,\theta }=\underset{\phi \neq \theta }{\sum }v_{x,y,\phi }^{mt}, \end{array}$$

where $v_{x,y,\phi }^{mt}$ is the activity of the MT neuron selective to direction ϕ. Introducing the inhibitory connections between neurons selective to different directions results in winner-take-all operation in the model. Apart from this inter-directional inhibition, there is another set of inhibitory connections between neighboring neurons selective to different directions. These inhibitory connections assist MT neurons in the propagation of activity to other regions by suppressing the activity of the neurons with different direction selectivities. These interactions are modeled by

(7) $$\begin{array}{l} \xi_{x,y,\theta }=\underset{\phi \neq \theta }{\sum }\underset{i\in \Phi }{\sum }\underset{j\in \Phi }{\sum }v_{x+i,y+j,\phi }^{mt}, \end{array}$$

where ϕ determines the spatial extent of these inhibitory connections.

**Figure 2:**
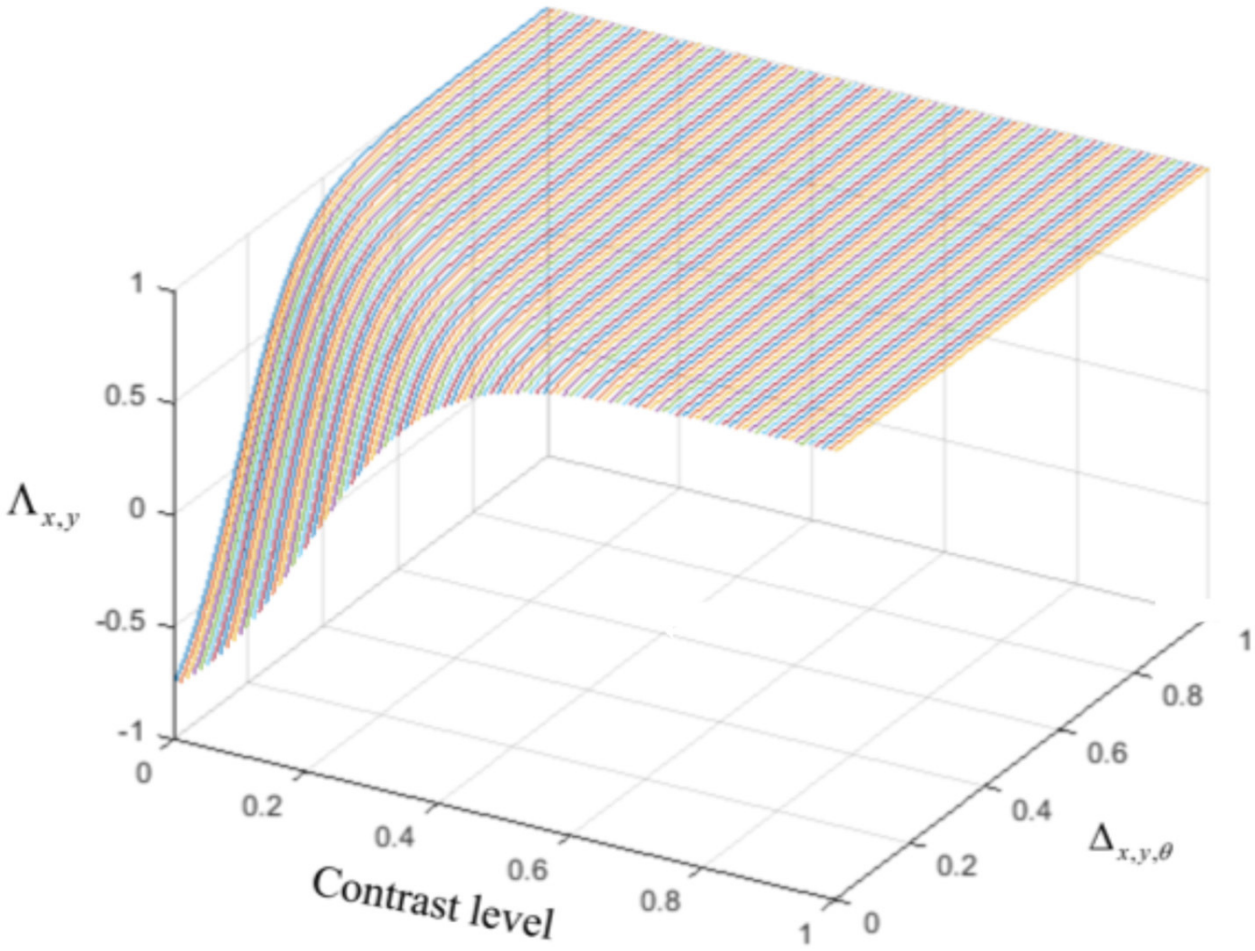
The level of the surround suppression depends on the contrast of the stimulus and the activities of the neurons selective to other directions. The negative values of χ_*x,y*,θ_ represent the excitatory effect of the surround and the positive values of χ_*x,y*,θ_ denote the inhibitory effect of the surround.

The last type of interconnection between neurons is defined by the interaction with each neuron's receptive field surround. The integrative surround of the MT neurons promotes the propagation of activity of MT neurons, while the antagonistic surround prevents the propagation of activity of MT neurons; i.e., where there is a discontinuity in the input stimulus resulting from the border of the stimulus or an overlap with another object. The level of coherency is determined using the form information provided by the activity of ECRF, which neurons that are sensitive to the luminance of the stimulus. The level of this contrast-dependent input (Figure 2) is computed as

(8) $$\begin{array}{l} \chi_{x,y,\theta }=kS\left(a\left(k\left(\Lambda_{x,y}-c^{r}\right)-1+b{△}_{x,y,\theta }+\alpha .\beta \right)\right)-1, \end{array}$$

where *S()* is a sigmoid function,

(9) $$\begin{array}{l} S\left(t\right)=\frac{1}{1+exp\left(-t\right)}, \end{array}$$

Λ_*x,y*_ indicates the level of the changes in the contrast of the stimulus, and △_*x,y,θ*_ is the discontinuity in the motion information occurring when there is another object moving in a different direction in the neuron's surround. The contrast level, Λ_*x,y*_, is computed by measuring the alterations in the levels of intensity of the neighboring pixels of the input image and normalizing this value by

(10) $$\begin{array}{l} \Lambda_{x,y}=\frac{\sum_{i}\sum_{j}|I_{x,y}-I_{x+i,y+j}|}{M}, \end{array}$$

where *I*_*x,y*_ is the intensity of the input image at location (*x, y*) and *M* is the spatial extent of the surround area of the MT neuron. _△*x,y*,θ_ is obtained as the result of the weighted summation of activities of complex V1 neurons selective to different directions and normalizing this value between 0 and 1,

(11) $$\begin{array}{l} \Delta_{x,y,\theta }=\frac{\sum_{i}\sum_{j}\left(\sum_{\psi }\left(1-cos\left(\psi -\theta \right)\right)v_{x+i,y+j,\psi }^{cx}\right)}{10M}, \end{array}$$

where *M* is spatial extent of the surround area of the neuron. The effectiveness weight of the activity of complex V1 neurons selective to the direction other than θ depends on the angular difference of the selective direction with θ.

The term, α, in equation (8) indicates the level of coherency in the input stimulus when the changes in the contrast level are below the threshold and is given by

(12) $$\begin{array}{l} \alpha =1-\left(\Lambda_{x,y}-c^{r}\right). \end{array}$$

The term, β, in equation (8) is the level of coherency of motion information across neurons selective to different directions, which is computed by

(13) $$\begin{array}{l} \beta =1-\Delta_{x,y,\theta }\text{.} \end{array}$$

Other parameters in equation (8) have constant values: *c*^*r*^ indicates the minimum of the contrast that triggers the suppressive surround, *k* limits the value of the level of surround suppression to saturate at 1, and *a* and *b* determine the slope of the changes in the level of suppression with contrast and the activity level of neurons with different direction selectivities.

The term, $k\left(\Lambda_{x,y}-c^{r}\right)-1$, within the sigmoid function represents the increase in the level of surround suppression where there is a high level of variation in the intensity of the input image. The term, *bΔ*_*x,y*,θ_, refers to an increase in the suppression level of the surround where the neighboring neurons selective to other directions are active. The term, α.β, models the integrative aspect of the surround where there is coherency in the input image and the neighboring neurons selective to other directions have a low level of activity while there is activity in the center. The surround of the neuron has a suppressive or integrative effect only when the center is activated by motion information. Therefore, the level of the surround effect is gated by the activity of the MT neuron at the center, described by

(14) $$\begin{array}{l} \chi_{x,y,\theta }=\{\begin{array}{c} \chi_{x,y,\theta \;if\;v_{x,y,\theta }^{mt}>0}\\ 0,\;otherwise \end{array} \end{array}$$

**Figure 3:**
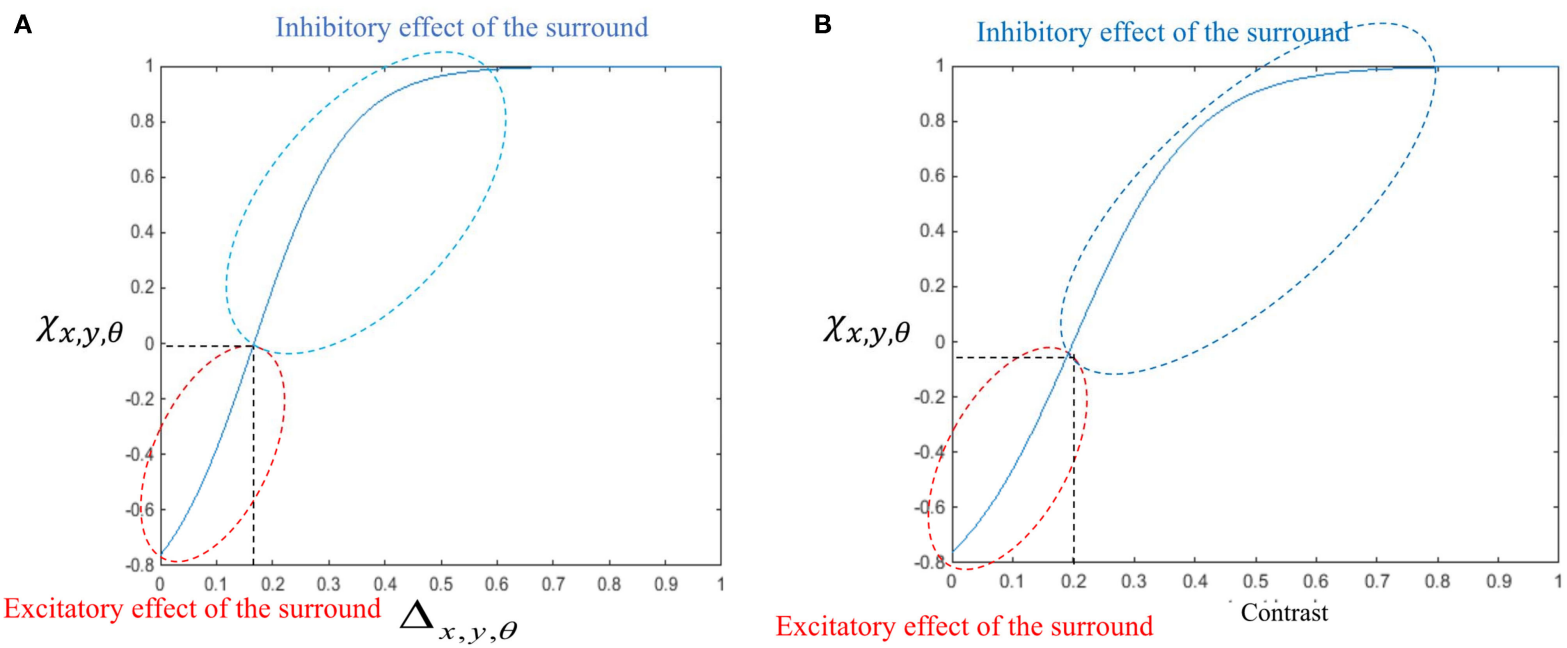
**(A)** The changes in the surround effect when the activity levels of the neurons selective to other directions change from 0 to 1 and the level of the contrast is 0. The effect of the surround is suppressive when the level of Δ_*x,y*,θ_ is above 0.14 but is integrative when these neurons have a low level of activity. **(B)** The changes in the level of surround suppression with contrast. The surround effect is suppressive when the contrast of the stimulus goes beyond the value of *c*^*r*^ = 0.2. The level of suppression increases with an increase in the contrast of the stimulus until it saturates. The surround effect is excitatory for low levels of the contrast, below *c*^*r*^.

The general influence of the surround suppression when there is motion in different directions upon the strength of the MT neuron activities, Δ_*x,y*,θ_, is a shift from 0 to 1, as shown in Figure 3A. Figure 3B shows the value of the surround suppression when the contrast level is changed at location (*x, y*), Λ_*x,y*_, from 0 to 1 when there is no motion detected at directions other than θ.

The general temporal dynamic behavior of component MT neurons is defined by

(15) $$\begin{array}{r} \frac{dv_{x,y,\theta }^{mt}\left(t\right)}{dt}=h(G_{mt}^{cx}v_{x,y,\theta }^{cx}\left(t\right)+G_{mt}^{es}v_{x,y,\theta }^{es}\left(t\right)\\ +G_{mt}^{mt1}\lambda_{x,y,\theta }\left(t\right)+G_{cs}^{mt}\kappa_{x,y,\theta }\left(t\right)-G_{mt}^{mt2}\gamma_{x,y,\theta }\left(t-T^{in}\right)\\ -G_{mt}^{mt3}\xi_{x,y,\theta }\left(t-T^{in}\right)-\tau_{mt}v_{x,y,\theta }^{mt}\left(t\right)-\chi_{x,y,\theta }\left(t\right)), \end{array}$$

where λ_*x,y*,θ_ is the excitation from neighboring MT neurons, κ_*x,y*,θ_ is excitatory input from standard V1 neurons gated by the activity of ECRF neurons, γ_*x,y*,θ_ is inter-directional inhibition, ξ_*x,y*,θ_ is long-range inhibition, and χ_*x,y*,θ_ is the effect of the surround on these neurons that depends on the coherency of the stimulus. These neurons receive excitatory input from V1 standard complex and end-stopped neurons, $v_{x,y,\theta }^{cx}$ and $v_{x,y,\theta }^{es}$, respectively. Finally, *h*() is a piece-wise linear saturation function that keeps the level of activity within a specified range (between 0 and 1),

(16) $$\begin{array}{l} h\left(x\right)=\{\begin{array}{ccc} 1,\;if\;x & \geq & 1\\ x,\;if\;0 & \leq & x\leq 1\\ 0,\;if\;x & < & 0 \end{array}\;. \end{array}$$

Table 3 summarizes the description and the role of each component in the equation 15.

**Table 3:** Description of the different components of equation 15.

**Component**	**Description**	**Contribution**
$G_{mt}^{cx}v_{x,y,\theta }^{cx}$	Input from standard complex V1 neurons	Provides initial motion information
$G_{mt}^{es}v_{x,y,\theta }^{es}$	Input from the end-stopped V1 neurons	Deal with the aperture problem
$G_{mt}^{mt1}\lambda_{x,y,\theta }$	Excitation from neighboring MT neurons	Propagation of the activity of the MT neurons
$G_{cs}^{mt}\kappa_{x,y,\theta }$	Interaction of ECRF neurons with standard complex V1 neurons	Discriminates the extrinsic terminators from the intrinsic terminators
$G_{mt}^{mt2}\gamma_{x,y,\theta }$	Inter-directional inhibition	Implements of the winner-take-all operation
$G_{mt}^{mt3}\xi_{x,y,\theta }$	Long range inhibition	Assists in propagation of the activity of the MT neurons
$\tau_{mt}v_{x,y,\theta }^{mt}$	Decay term for the activity of the neurons	Low-pass filtering due to membrane dynamics
χ_*x,y*,θ_	The effect of the surround of the MT neurons	Modulatory effect of the surround

There is explicit evidence of the existence of end-stopped V1 neurons (Hubel and Wiesel, 1965; Pack et al., 2003; Tsui et al., 2010). According to the findings of Movshon and Newsome (1996) these neurons project to area MT, which we have incorporated into our model. There is also evidence for the existence of the orientation selective V1 neurons that are contrast sensitive and have suppressive surrounds (Sceniak et al., 2001). These neurons are included as ECRF neurons in our model and their role in motion detection of the moving stimuli is described. There are also neurophysiological experiments showing the center-surround interactions of the MT neurons and the input that they receive from special complex V1 neurons. The modulatory surround effects of the MT neurons are shown in the neurophysiological experiments by Huang et al. (2007, 2008). However, the circuitry of this adaptive modulatory effect is unknown. A possible mechanism for this adaptive modulatory effect of the center-surround interaction in MT is proposed in our model (Table 4).

**Table 4:** A summary of the known neurophysiological findings and unknown features that are hypothesized in the model.

**Existence of different subtypes of neurons/ connections/ circuitry of the mechanisms/ phenomena**	**Known**	**Selected references**
Direction selective standard complex V1 neurons	✓	Hubel and Wiesel (1962); Dreher (1972); Movshon (1975); Movshon et al. (1978a,b)
End-stopped V1 neurons	✓	Hubel and Wiesel (1965); Sceniak et al. (2001); Pack et al. (2003); Tsui et al. (2010)
Orientation selective V1 neurons with suppressive surround (ECRF neurons)	✓	Cavanaugh et al. (2002)
Component and pattern selective MT neurons	✓	Adelson and Movshon (1982); Albright (1984); Rodman and Albright (1989); Livingstone et al. (2001); Born and Bradley (2005)
Difference in the temporal dynamics of the component and pattern MT neurons	✓	Smith et al. (2005, 2009)
Projection of the complex V1 neurons to MT area	✓	Maunsell and van Essen (1983); Movshon and Newsome (1996)
Projection of the end-stopped V1 neurons to MT area	✓	Movshon and Newsome (1996); Sceniak et al. (2001)
Projection of the ECRF neurons to MT area		Hypothesized in the model
Suppressive effect of the surround in V1	✓	Hubel and Wiesel (1968); Cavanaugh et al. (2002)
Center-surround interaction of MT neurons	✓	Allman et al. (1985); Raiguel et al. (1995); Albright and Stoner (2002); Born and Bradley (2005)
Adaptive modulatory effect of the surround	✓	Huang et al. (2007, 2008)
Circuitry of the modulatory effect of the surround		Hypothesized in the model
Contrast dependency of the pattern selectivity of the MT neurons	✓	Kumbhani et al. (2008)
Contrast dependency of the suppressive effect of the surround in MT neurons	✓	Pack et al. (2005)
The activity of V1 neurons is gated by the activity of the ECRF neurons		Hypothesized in the model
The level of the excitatory connections between MT neurons depends on ECRF V1 neurons		Hypothesized in the model

## Results

**Figure 4:**
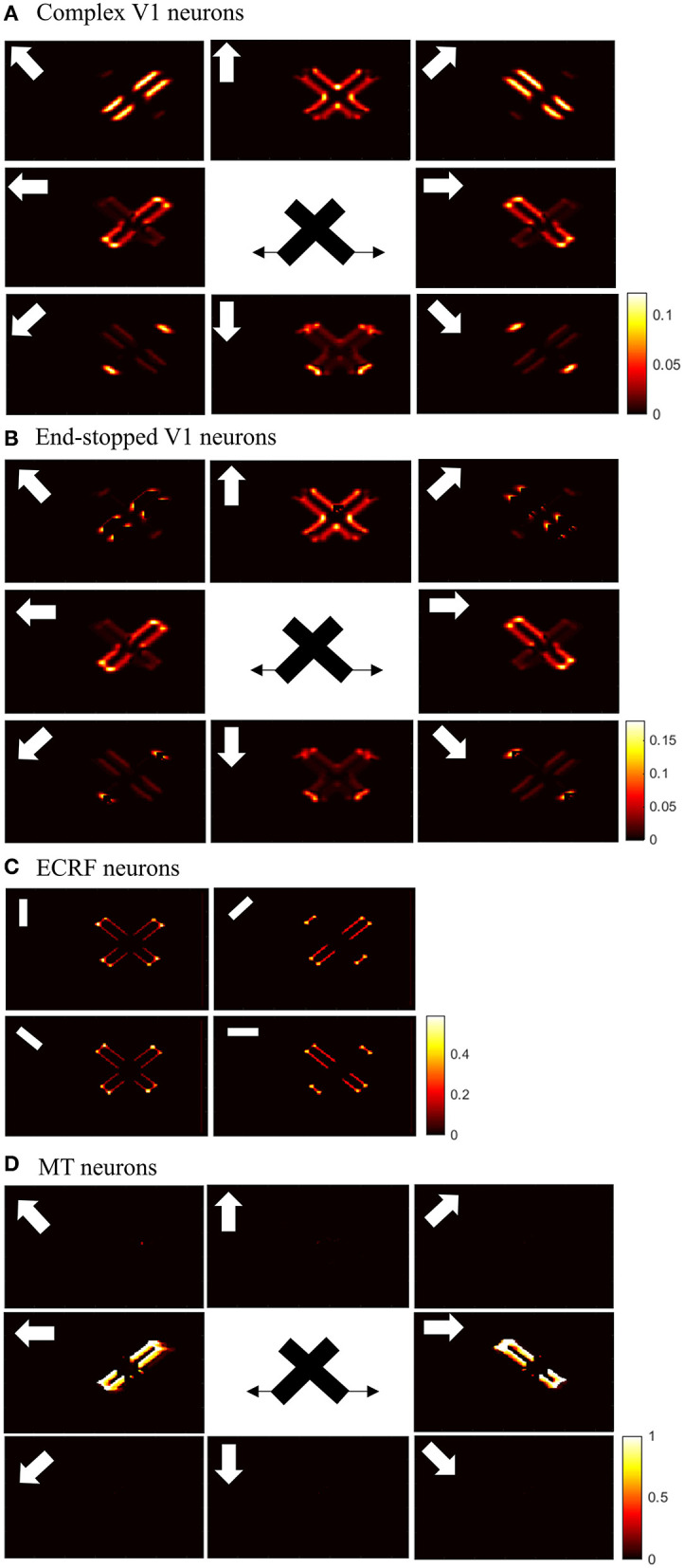
**(A)** The activities of standard complex V1 neurons selective to eight different directions of motion in response to overlapping moving bars with the same contrast. Each box shows the activities of the neurons selective to the direction shown by the white arrow. The axes represent the spatial location. The color bar shows the strength of the activity, brighter for higher values. The white arrows indicate the preferred direction of the neurons in each graph. The stimulus is two crossing bars with the same level of contrast, as illustrated in the middle of the figure. The bar with 135° orientation is moving to the right and the bar with 45° orientation is moving to the left (black horizontal arrows). The neurons have high levels of activity at the terminators and along the edges of the bars. **(B)** The activities of end-stopped V1 neurons. The neurons have high levels of activity at both the intrinsic and extrinsic terminators. **(C)** The activities of ECRF V1 neurons. The preferred orientations of the neurons in each graph are shown by the white bars. The neurons have the highest levels of activity at the intrinsic terminators and their activities are strongly suppressed at the extrinsic terminators. **(D)** The activities of MT neurons with adaptive surrounds. The MT neurons selective to the rightward direction have high levels of activity in response to the motion of the bar moving to the right and the neurons selective to the leftward direction have high levels of activity in response to the motion of the bar moving to the left. The activities of the MT neurons are suppressed at the crossing junction where there is more than one moving object in the same depth plane.

Initial motion signals are extracted by standard complex neurons in V1. The small receptive fields of these neurons result in ambiguous motion information. The activities of these neurons in response to two overlapping moving bars with the same contrast are shown in Figure 4A. The neurons selective to directions perpendicular to the edges of the bars have high levels of activity at these locations because of the aperture problem. The activities of neurons at the intrinsic terminators express accurate estimations of the directions of movement of the bars. The neurons selective to the upward direction also display high levels of activity at the extrinsic terminators formed at the crossing points of the bars.

The existence of end-stopped neurons is essential for MT neurons to differentiate unambiguous motion information of the terminators from the ambiguous motion information that arises from the aperture problem. Figure 4B shows the activities of the end-stopped neurons in response to the two overlapped moving bars. Among the neurons selective to the rightward direction, those at the intrinsic terminators of the bar that is moving to the right, have high levels of activity. Similarly, among the neurons selective to the leftward direction, the end-points of the bar that is moving to the left, have high levels of activity. Apart from the intrinsic terminators with unambiguous motion information, end-stopped neurons selective to upward directions have higher levels of activity at the extrinsic terminators, which represent upward local motion of the crossing junction of the overlapping bars.

To discriminate the motion signals of the intrinsic from the extrinsic terminators, the third set of luminance sensitive V1 neurons with suppressive surrounds play a significant role. The activities of these neurons are shown in Figure 4C. They are strongly suppressed at the extrinsic terminators where the inhibitory surrounds of the neurons are more stimulated compared to the intrinsic terminators. Therefore, excitatory connections from these neurons assist the modeled MT neurons to differentiate the unambiguous motion signals of the intrinsic terminators from the local motion signals of the extrinsic terminators.

The activities of the three sets of V1 neurons are transmitted to the neurons in MT. The surrounds of the MT neurons have an excitatory effect when there is motion coherency. The excitatory effects of the surround assist MT neurons to propagate motion signals. The surrounds of the neurons have an inhibitory effect at the discontinuities where the contrast level is high, which assist MT neurons to segregate the motion signals of different moving objects. The disambiguation of the motion signals by MT neurons takes 30 ms. Figure 4D shows the activities of MT neurons. MT neurons represent the correct direction of the motion of the overlapping bars. The MT neurons selective to the rightward direction have high levels of activity along the edges of the bar moving to the right and the MT neurons selective to the leftward direction have high levels of activity along the edges of the bar that is moving to the left. Without the contrast adaptive property of the MT neurons, the model requires two sets of MT neurons, named integration and segmentation MT neurons, for the correct estimation of motion. Introducing the contrast adaptive feature of the MT neurons, which also accords with existing neurophysiological findings, creates a unified model of MT neurons.

**Figure 5:**
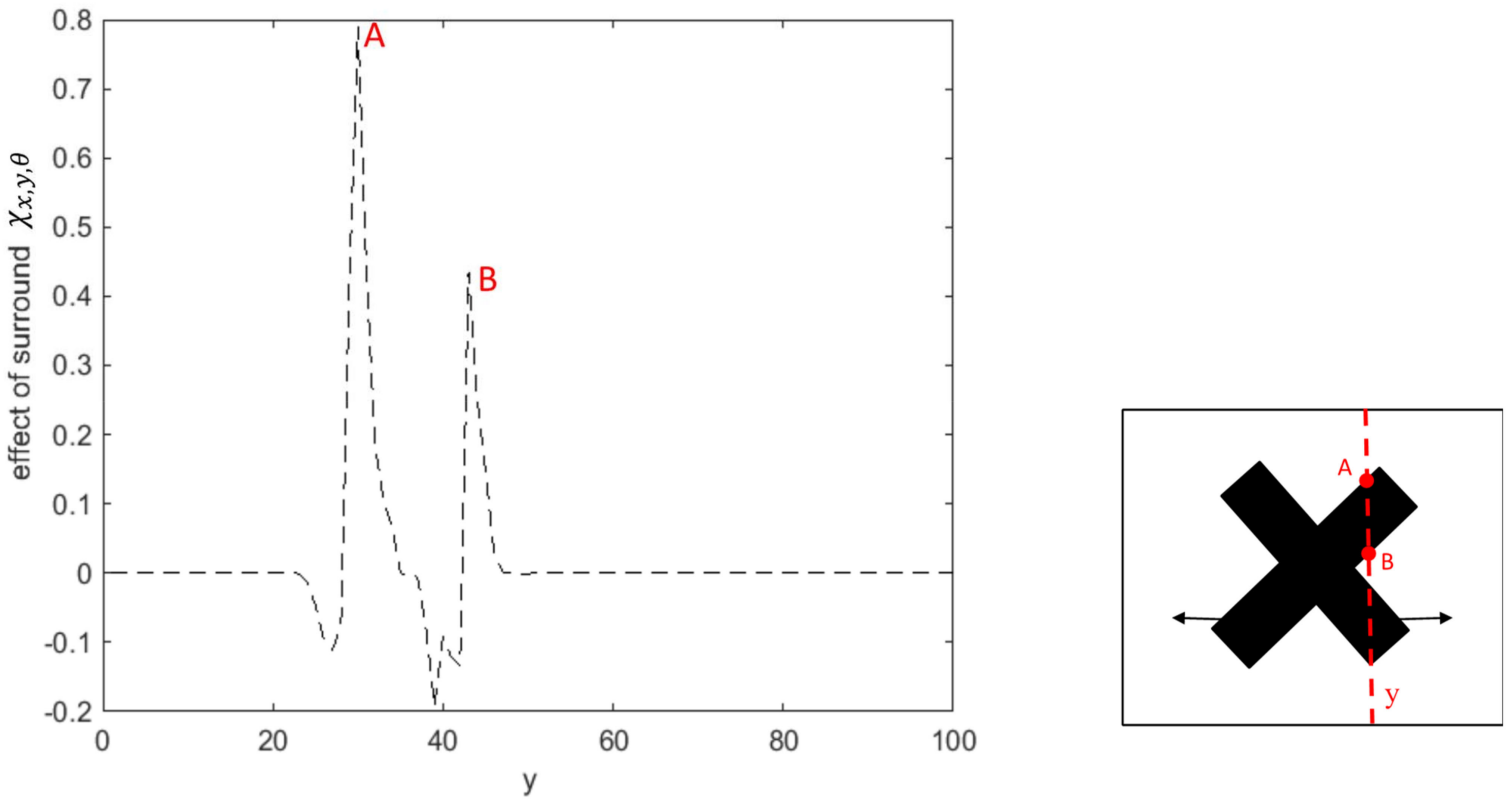
The surround effect of the MT neurons selective to the leftward direction with receptive fields along the vertical axis of the stimulus. The neurons have a suppressive surround at the discontinuities along the edge of the leftward moving bar. The neurons have an excitatory effect followed by a suppression that results in the propagation of the activity of the neurons responding to the intrinsic terminators. The surround of the neurons selective to the leftward direction are not activated along the edge of the bar, which is moving to the right.

The spatial dynamics of the surround effect of the MT neurons selective to the leftward direction is shown in Figure 5. The surround regions of the neurons are suppressive at the sharp edges of the bar, which is moving to the left. The surrounds of the neurons selective to the leftward direction are not activated along the edge of the bar, which is moving to the right. The inhibitory effect of the surround switches to the excitatory effect along the bar to assist MT neurons in propagating the motion information from the intrinsic terminators.

**Figure 6:**
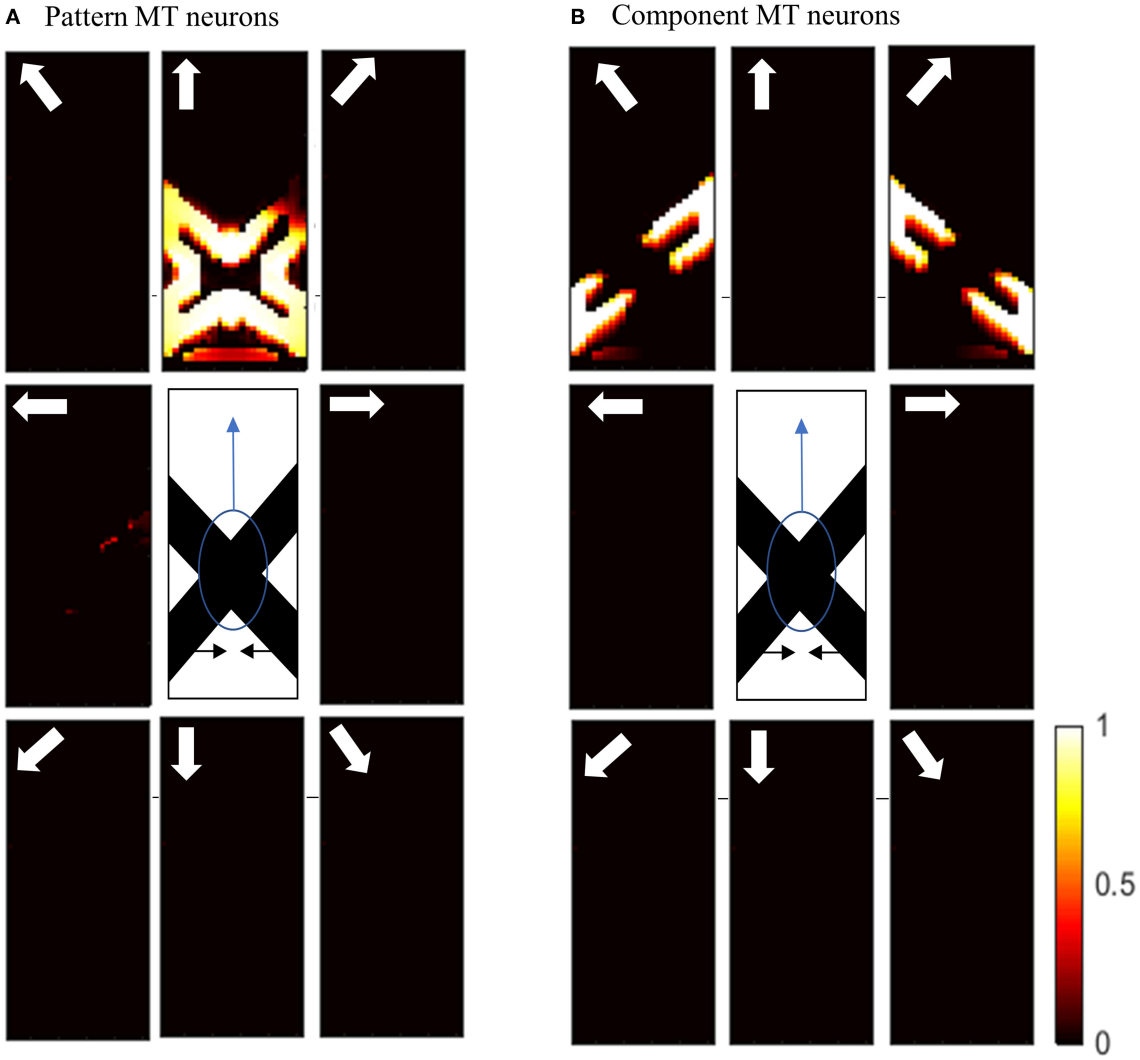
**(A)** The activities of pattern motion selective MT neurons responding to pattern motion. The neurons selective to the upward direction have the highest levels of activity, representing the pattern motion of the stimulus. The stimulus is shown in the middle, which is two crossing bars with hidden intrinsic terminators. The bars are actually moving to the left and right (horizontal black arrows). Each box shows the activities of the neurons selective to the direction shown by the white arrow. The color bar shows the strength of activity (brighter for higher values) and the axes represent the spatial location. **(B)** The activities of the neurons selective to the component motion in response to the pattern motion. The neurons selective to the up-right and up-left directions have the highest levels of activity, representing the component motion of the stimulus.

To investigate the pattern motion selectivity of MT neurons, we examine the response of the model to a plaid pattern, which is obtained by occluding the intrinsic terminators of the overlapping moving bars. The responses of the MT neurons to the pattern or component motion of the stimulus are highly dependent on the connection strengths of the received inputs computed by standard V1 and ECRF neurons. The activities of the MT neurons with strong connections associated with the interaction of ECRF and standard V1 neurons are shown in Figure 6B.

The interaction with the ECRF neurons prevents the propagation of motion information from the extrinsic terminators along the whole of the stimulus. Therefore, MT neurons respond to the component motion information that they receive from standard V1 neurons and their response does not change over time, in contrast to the pattern MT neurons. The MT neurons respond to the pattern motion of the stimulus when there is a weak connection from the ECRF neurons. The activities of the neurons responding to the pattern motion of the stimuli are shown in Figure 6A.

The motion information of the extrinsic terminators is not suppressed when there is a weak connection from ECRF neurons to the pattern MT neurons. Therefore, the activities of the neurons in response to the local motion of the extrinsic terminators propagate to the other regions. Pattern MT neurons represent the component motion of the stimulus at the beginning and they respond to the pattern motion after a delay. This is because of the time required for the propagation of the motion information from extrinsic terminators.

**Figure 7:**
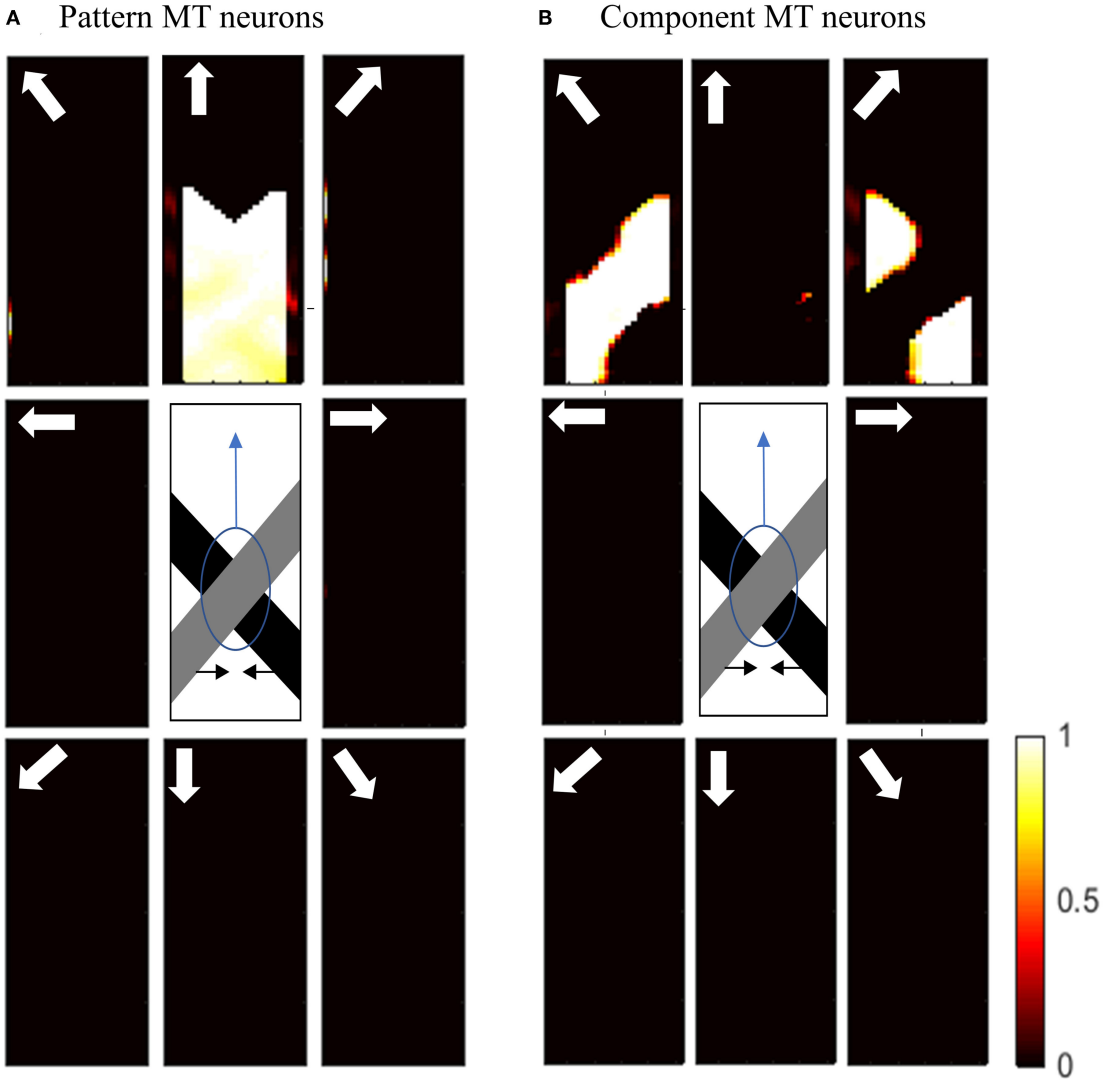
**(A)** The activities of pattern motion selective MT neurons responding to pattern motion with bars of different contrasts. The neurons selective to the upward direction have the highest levels of activity, representing the pattern motion of the stimulus. The stimulus is shown in the middle, which is two crossing bars with hidden intrinsic terminators. The bars are actually moving to the left and right (horizontal black arrows). Each box shows the activities of the neurons selective to the direction shown by the white arrow. The color bar shows the strength of activity (brighter for higher values) and the axes represent the spatial location. **(B)** The activities of the neurons selective to the component motion in response to the pattern motion. The neurons selective to up-right and up-left directions have the highest levels of activity, representing the component motion of the stimulus.

Figure 7A shows the activities of pattern MT neurons responding to the pattern motion in the case of a difference in the contrast of the bars. The MT neurons selective to the upward direction have a high level of activity in response to the upward pattern motion. Figure 7B shows the activities of component MT neurons in response to the pattern motion. The neurons are able to detect the individual component motions and, as the contrasts of the bars are different, they are also able to estimate which bar is moving in front.

## Discussion

We developed a model of MT neurons that adapts its properties depending on the input stimulus. The activities of these neurons are influenced by their ECRFs, which have directionally antagonistic or facilitatory effects depending on the properties of the input stimulus. For preferred direction motion, the surround regions are suppressive if there are discontinuities in the visual field or the contrast levels of the stimulus are high. The amount of suppression increases with increases in contrast. The surrounds are facilitatory when there is coherency in the directions of motion across the visual field, which assists the modeled MT neurons in the propagation of motion signals. Our model explains the circuitry of this modulatory effect of surrounds and provides insights into the mechanism by which different surround effects arise depending on the properties of the input stimulus.

The modulatory effect of the surround of MT neurons has been reported in several neurophysiological findings. For example, the neurophysiological experiments by Huang et al. (2007) showed that integration-MT neurons switch to segmentation-MT neurons depending on the ambiguity in the motion information of the stimulus. According to their findings, MT neurons are able to overcome the aperture problem by being integrative but can also code segmentation when stimulated by random dot patterns (Huang et al., 2007, 2008). The psychophysical studies by Tadin et al. (2003) also provide some insights on the possibility of the contrast dependency of the surround. The adaptive change of the relationship between the center and surround regions of MT neurons with contrast has also been shown by other studies. The experiments by Pack et al. (2005) showed a reduction in the suppression level as stimulus contrast was decreased.

The surrounds of the MT neurons in our model are contrast adaptive. Therefore, they have the characteristics of integration MT neurons when there is coherency in the input stimulus to facilitate the propagation of motion signals from bar terminators to other regions. The surrounds of the neurons have a suppressive effect in the case of high contrast at motion discontinuities. The pattern or component selectivity of the MT neurons depends on the input that they receive from the interaction of the ECRF neurons and standard complex V1 neurons. The strong connections from these neurons provide form information to assist MT neurons in suppressing the effects of the extrinsic terminators and respond to the component motions of the stimuli. Weak connections from these neurons result in pattern motion selectivity of the MT neurons. The pattern selectivity of the MT neurons is highly dependent on the contrast of the stimulus. The pattern motion selectivity index drops significantly when the contrast of the overlapping bars differs. The difference in the contrast of the overlapping bars results in the formation of illusionary depth when it appears that one of the bars is sliding over the other bar, which results in the dominance of component over pattern motion selectivity. The experiments by Kumbhani et al. (2008) supports the effect of the contrast on the pattern selectivity of the neurons. The results of their experiments show that the pattern selectivity index of the MT neurons drops significantly if the contrast of one of the gratings is reduced (Kumbhani et al., 2008).

In accordance with these neurophysiological findings, MT neurons are not categorized into integration or segmentation neurons in our proposed model. They represent the integrative or segregated features of the MT neurons depending on the characteristics of the input stimulus. Therefore, the activities of the MT neurons are influenced by their adaptive surround, which changes over time and space depending on the stimuli. Also, our model suggests that MT neurons are not grouped into different types of pattern or component MT neurons. Their level of pattern selectivity is only determined by the strength of the connections from ECRF neurons. Therefore, they are selective to the component motions of the stimuli when they receive strong input from ECRF neurons, and they represent the feature of the pattern selective MT neurons when the strength of the connection from the form processing input is weak or there is no connection. This theory on the pattern motion selectivity of the MT neurons contrasts with the modeling works by Simoncelli and Heeger (1998) and Rust et al. (2006). Simoncelli and Heeger (1998) suggested a linear-nonlinear model, which was further developed by Rust et al. (2006). The non-linear normalization in these models suppresses the unambiguous motion signals resulting from the aperture problem, which is similar to the role of end-stopping neurons in our model. The mechanism for pattern motion detection in the linear-nonlinear model is based on the hierarchical relationship between component and pattern MT neurons. Our model suggests an entirely different mechanism for pattern motion selectivity of MT neurons, which can explain the spatial and temporal limits on pattern motion selectivity of MT neurons observed in the experiments by Majaj et al. (2007) and Kumbhani et al. (2015). However, the linear-nonlinear model is not capable of explaining these features of pattern MT neurons. Due to the simulation of the propagation of activity, our model can replicate the temporal dynamics of component and pattern MT neurons observed by Smith et al. (2005, 2009), which shows a temporal delay in the pattern motion detection of the MT neurons. The linear-nonlinear model lacks these properties of the MT neurons (Simoncelli and Heeger, 1998; Rust et al., 2006).

## Data Availability Statement

All datasets generated for this study are included in the article/supplementary material.

## Author Contributions

PZ, TK, MI, AB, and DG: conceptualization, methodology, validation, and writing–review and editing. TK, MI, AB, and DG: funding acquisition and supervision. DG: project administration and resources. PZ: investigation, software, visualization, formal analysis, and writing–original draft. All authors contributed to the article and approved the submitted version.

## Conflict of Interest

The authors declare that the research was conducted in the absence of any commercial or financial relationships that could be construed as a potential conflict of interest.
